# Crazy‐paving patterns as rare radiological manifestations of pulmonary cryptococcosis: a case report

**DOI:** 10.1186/s12890-021-01450-5

**Published:** 2021-03-12

**Authors:** He Yu, Kaige Wang, Dong Huang, Lu Wen, Ying Zhang, Ye Wang, Yongjiang Tang, Jiajia Dong, Zongan Liang

**Affiliations:** 1 Department of Respiratory and Critical Care Medicine, West China Hospital, Sichuan University, No 37 Guoxue Alley, Chengdu, 610041 Sichuan China; 2 Department of Respiratory and Critical Care Medicine, The People’s Hospital of Pengzhou, Chengdu, Sichuan China; 3 Department of Pathology, West China Hospital, Sichuan University, Chengdu, Sichuan China

**Keywords:** Crazy‐paving pattern, Pulmonary cryptococcosis, Primary ciliary dyskinesia, Case report

## Abstract

**Background:**

Crazy-paving patterns are rarely reported as radiological manifestations of pulmonary cryptococcosis.

**Case presentation:**

Herein, we presented a very rare case of a crazy-paving pattern as a radiological manifestation of pulmonary cryptococcosis in a patient with primary ciliary dyskinesia. The diagnosis of pulmonary cryptococcosis and primary ciliary dyskinesia was ultimately confirmed by bronchoscopic biopsy, fungus culture, whole exome sequencing of blood, etc. The patient received flucytosine (PO, 5 g per day) and amphotericin B (IV, 70 mg per day) during hospitalization and sequential therapy with voriconazole (PO, 200 mg twice a day) after discharge. He recovered during follow-up.

**Conclusions:**

We concluded that pulmonary cryptococcosis should be considered a possible cause of crazy-paving patterns in chest CT scans.

## Background

Pulmonary cryptococcosis is a common opportunistic infectious disease [1]. The crazy-paving pattern refers to areas of ground-glass attenuation associated with inter- and intralobular septal thickening on chest high-resolution CT (HRCT) [2]. However, it has rarely been reported as a radiological manifestation of pulmonary cryptococcosis. We herein presented a very rare case of a crazy-paving pattern as a radiological manifestation of pulmonary cryptococcosis in a patient with primary ciliary dyskinesia.

## Case presentation

**Fig. 1:**
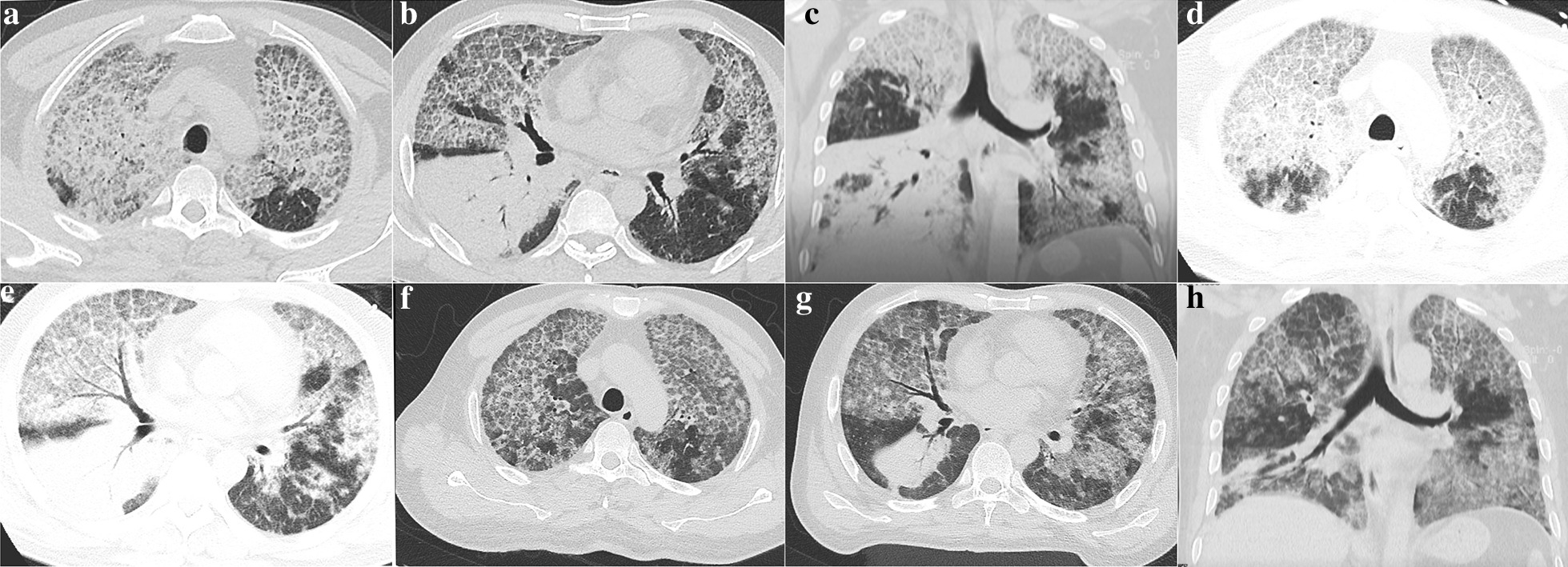
**a**–**c** (chest HRCT, June 23, 2020): bilateral, diffuse reticular pattern and ground-glass opacity (crazy-paving pattern) in the right upper and middle lobes and left upper lobe; exudation shadow in the left lower lobe; patchy consolidation in the right lower lobe. **d**, **e** (chest CT, July 13, 2020): exudation shadow in right upper and left upper lobes decreased; exudation shadow in right middle and left lower lobes increased; patchy consolidation in right lower lobe decreased slightly. **f**–**h** (chest HRCT, September 21, 2020): crazy-paving pattern in right upper and middle lobes and left upper lobe, and patchy consolidation in right lower lung both decreased significantly

The patient was a 46-year-old male construction worker. He presented to our hospital with complaints of cough with a small amount of white sputum without obvious cause for three months and with fever and dyspnea for six days. There were no complaints of chills or chest pains. He had been administered cefoperazone-sulbactam at another institution a few days prior to admission at our hospital. However, the therapeutic effect was unsatisfactory. He had been diagnosed with secondary bilateral pulmonary tuberculosis at our institute four years ago. After receiving regular anti-tuberculosis treatments with “2HREZ/10HRE”, his symptoms of cough and shortness of breath improved. Since then, the patient failed to participate in follow-up. There was no history of possible poultry exposure, smoking or drinking. On admission, he was conscious. The physical examination revealed a respiratory rate of 28 breaths/min. Lung percussion of the right lower lobe showed dullness and flatness. On lung auscultation, weak breath sounds were heard in the right lower lobe, and diffuse bilateral moist rales were also heard. A complete laboratory examination was performed, and the significant laboratory findings were as follows. The white blood cell level was 13.05 × 10^9^/L, and the percentage of neutrophils was 88.3%. The level of procalcitonin was 0.21 g/ml, and the PaO_2_/FiO_2_ ratio was 115 mmHg during noninvasive mechanical ventilation. The carcinoembryonic antigen level was 14.7 ng/ml, and the CD4 T cell count was 48 cells/µl. The test for human immunodeficiency virus infection was negative. Anti-nuclear antibodies, anti-extracted nuclear antigen (ENA) autoantibodies, myositis-specific antibodies, and anti-neutrophil cytoplasmic antibodies (ANCAs) were all negative. Chest HRCT (Fig. 1a–c) revealed a bilateral, diffuse reticular pattern accompanied by ground-glass opacity, which was called a “crazy-paving pattern”. Furthermore, patchy consolidation was found in the right lower lobe. There was no pulmonary nodule, pleural effusion or mediastinal lymphadenopathy. After admission, the patient was given moxifloxacin (400 mg, IV, once a day), methylprednisolone (40 mg, IV, twice a day) and a noninvasive ventilator.

**Fig. 2:**
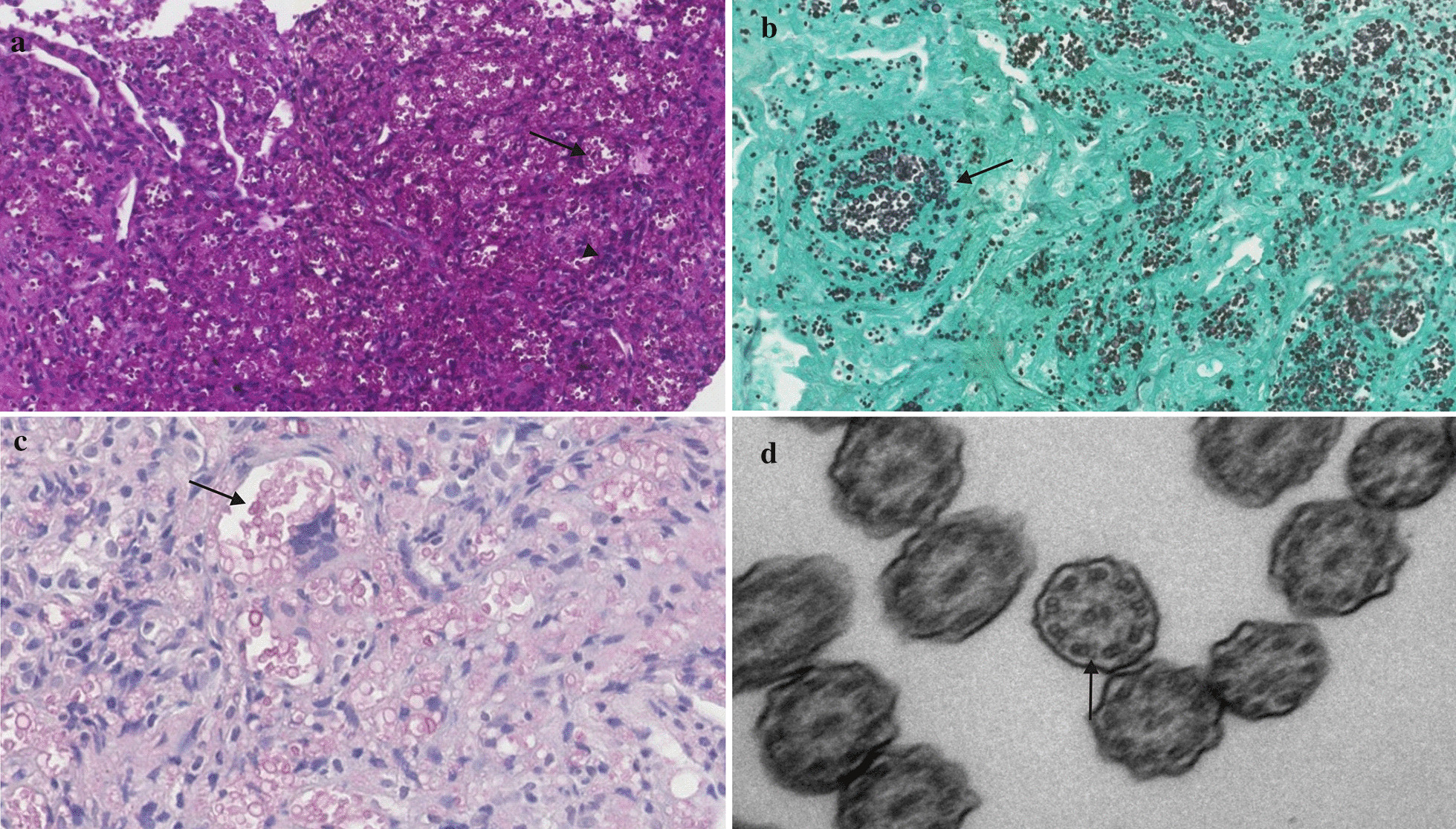
**a**–**c** Staining results. **a** PAS (+), 200×; **b** Silver staining (+), 200×; **c** Mayer’s dyeing (+), 400×. Meanwhile, pathologic examination showed proliferation of alveolar epithelial cells and diffuse infiltration of foam cells, inside of which a large number of spherical fungi were found. According to these results, the diagnosis of pulmonary *Cryptococcus neoformans* infection was established. **d** Electron microscopic examination showed bronchociliary outer dynein arm partial defects

After 5 days of antibiotic treatment, his symptoms did not improve. As a result, further examinations were performed. The test of serum cryptococcal antigen was positive, and the titer of cryptococcal antigen was greater than 1:2560. The patient underwent bronchoscopy, and bronchoalveolar lavage fluid (BALF) from the right upper lobe was obtained. The BALF was clear and bright in appearance. Periodic acid-Schiff (PAS) staining showed a small number of suspected positive protein-like bodies. The alcian blue staining result was negative. Next-generation sequencing (NGS) of the bronchoalveolar lavage fluid showed *Cryptococcus neoformans* with 3605 reads. Consistently, fungal culture of the BALF showed the growth of *Cryptococcus neoformans*, which was sensitive to fluconazole. Nucleic acid testing of *Pneumocystis jiroveci* from the BALF showed negative results. To further confirm the diagnosis, a CT-guided percutaneous lung biopsy was performed. Pathologic examination showed the proliferation of alveolar epithelial cells and diffuse infiltration of foam cells, inside of which a large number of spherical fungi were found (Fig. 2a–c). The staining results were as follows: silver staining (+), PAS (+), Mayer’s dyeing (+), d-PAS (+), and acid fast staining (−). According to these results, the diagnosis of pulmonary *Cryptococcus neoformans* infection was established. Furthermore, a lumbar puncture was performed to check whether the central nervous system was involved. The cerebrospinal fluid (CSF) was essentially normal. The pressure of the CSF was 145 mm H_2_O. The CSF was colorless and transparent in appearance, and no nucleated cells, red blood cells or pus cells were found. Biochemical tests of the CSF included trace protein: 0.43 g/L, chlorine: 119 mmol/L, and glucose: 4.58 mmol/L. The ink stain of the CSF was negative. Magnetic resonance imaging (MRI) of the head presented normal results. Based on the above results, the anti-infective treatments were changed to fluconazole (600 mg, IV, once a day) and piperacillin-tazobactam (4.5 g, IV, every 8 h).

However, after 20 days of antifungal therapy, the patient still suffered from respiratory distress. Additionally, the PaO_2_/FiO_2_ ratio did not increase significantly. A repeated chest CT scan was performed (Fig. 1d, e), which indicated that the patchy consolidation had decreased but the bilateral reticular pattern had increased significantly. The patient underwent new bronchoscopy after informed consent was obtained. Lung biopsy of the right upper lobe revealed proliferation of alveolar epithelial cells and interstitial fibroblasts, diffuse inflammatory infiltration, and a broken bronchial mucosal epithelium. The second fungal culture of the BALF showed *Cryptococcus neoformans*, which was resistant to fluconazole but sensitive to 5-fluorocytosine and amphotericin B. The titer of the Cryptococcus antigen was still greater than 1:2560. To identify underlying comorbidities and possible risk factors for pulmonary cryptococcosis, further bronchoscopic biopsy of the bronchial mucosa was also completed. Electron microscopic examination showed bronchociliary outer dynein arm partial defects (Fig. 2d). There were some significant findings of whole exome sequencing of the blood: heterozygous mutations in the DNAH5 gene (EX66, change in DNA: NM_001369.2: c.11350G>A, change in amino acids: P. Va13784Met; EX21, and change in DNA: NM_001369.2: c.3234T>C, change in amino acids: p. Asp1078Asp). The patient did not have any other features or positive findings of primary ciliary dyskinesia. His mother died of hypertension and stroke, and his father was still living and in good health. His sons were both healthy without any known pulmonary diseases.

Finally, the patient was diagnosed with the following: (1) severe pulmonary cryptococcosis; (2) type I respiratory failure; and (3) primary ciliary dyskinesia. Patient treatments mainly included infection control and respiratory support. The antifungal therapies were also adjusted to flucytosine (PO, 5 g per day) and amphotericin B (IV, 70 mg per day) according to the results of drug sensitivity analysis. Two weeks later, the treatment was changed to voriconazole (PO, 200 mg, twice a day). Additionally, the noninvasive ventilator was gradually changed to a high-flow nasal cannula (HFNC). Finally, the patient was discharged after significant improvements in oxygenation.

Two months after discharge, the patient underwent follow-up. The chest HRCT scan showed significant improvement (Fig. 1f–h): the patchy consolidation in the right lower lobe and crazy-paving pattern in the bilateral upper lobes decreased significantly. Moreover, the CD4 T cell counts also returned to normal (540 cell/µl). The flow of diagnosis and treatments of the patient is shown in Table 1.

**Table 1:** Flow of diagnosis and treatments

Indexes	Normal range	Hospitalization						Discharged from hospital
		Day 1	Day 5	Day 10	Day 20	Day 35	Day 40 (discharge)	40 days after discharge
White blood cell count (×10^9^/L)	3.5–9.5	13.05	10.07	17.77	23.85	7.42	6.94	5.36
Neutrophil (%)	40–75	88.3	92.4	83.2	96.9	82.2	76.3	57.3
CD4 T cell count (cell/µL)	471–1220	48	–	–	115	–	316	434
PCT (ng/mL)	0–0.05	0.21	0.07	0.1	0.61	0.11	0.05	–
PaO2/FiO2 ratio (mmHg)	400–500	115	110	105	110	160	240	360
Etiological examination			NGS (BALF): *Cryptococcus neoformans*; serum cryptococcal antigen: positive; lung biopsy: cryptococcus		fungus culture of BALF: *Cryptococcus neoformans*, resistant to fluconazole			
Image		Figure 1a–c			Figure 1d, e			Figure 1f–h
Drugs		Moxifloxacin: 400 mg qd; methylprednisolone: 40 mg bid	Fluconazole: 600 mg qd; piperacillin-tazobactam: 4.5 g q 8 h		Flucytosine: 5 g qd PO; amphotericin B: 70 mg qd		Voriconazole: 200 mg bid PO	Voriconazole: 200 mg bid PO
Respiratory support		Noninvasive ventilator				HFNC	nasal cannula	

*NGS* next-generation sequencing, *BALF* bronchoalveolar lavage fluids, *HFNC* high-flow nasal cannula

## Discussion and conclusions

We reported the first case of a crazy-paving pattern as a radiological manifestation of pulmonary cryptococcosis. Detailed etiological examination ruled out infections from viruses, pneumocystis or other pathogens. The bronchoscopy results were inconsistent with pulmonary alveolar proteinosis. Considering previous infection with *Mycobacterium tuberculosis* and current severe *Cryptococcus neoformans* pneumonia, we suspected that the patient might be immunocompromised. This speculation was finally verified by electron microscopic examination and whole exome sequencing of the blood.

A crazy-paving pattern was first used to describe the imaging characteristics of pulmonary alveolar proteinosis. Currently, this manifestation can be identified in pulmonary edema, nonspecific interstitial pneumonia, pulmonary hemorrhage, sarcoidosis, viral infection, peripheral T-cell lymphoma, invasive mucinous adenocarcinoma, etc. [2–6]. Cryptococcosis has become a common opportunistic infectious disease due to several factors, such as climate change, the evolution of *Cryptococcus*, and the growth of the pathogen in a number of susceptible populations [7]. The most common transmission of *Cryptococcus* causing infection is via inhalation. Spores of *Cryptococcus* may reach the lower airways and pulmonary alveoli. Respiratory defenses such as mucociliary transport do not prevent these spores from reaching the distal alveoli. [8] The central nervous system and lung are the most commonly involved and infected organs. In most cases, the symptoms of pulmonary cryptococcosis are nonspecific, including cough, expectoration, chest tightness, fatigue, etc. The patients’ conditions might vary from asymptomatic to severe acute respiratory distress syndrome (ARDS) [9]. It should be noted that the severity and radiological features of pulmonary cryptococcosis largely depend on the immune status of the patient. Symptoms of immunocompetent patients are often mild or even absent. However, immunocompromised patients often have extensive and severe symptoms or even disseminated cryptococcosis. Patients might develop fever, shortness of breath, hypoxemia and acute respiratory failure in a short time [10]. For chest CT scans, localized solitary or multiple peripheral nodules or pneumonia-like lesions are the most common imaging characteristics in immunocompetent patients. Diffuse pneumonia-like infiltration and consolidation with cavities and “halo signs” are more likely to be found in immunocompromised patients [11].

Primary ciliary dyskinesia is a rare ciliopathic genetic disorder that causes defects in cilia action. DNAH5 is a main ciliary dyskinesia gene, and mutations of DNAH5 have been identified in 15 % of primary ciliary dyskinesia patients [12]. The heterozygous mutations in the DNAH5 gene that occurred in EX66 and EX21 in our patient have rarely been reported previously. Abnormal expression of mutated DNAH5 proteins leads to outer dynein arm defects and decreased cilia movement. As a result, patients experience recurrent airway infections or systemic opportunistic infections [13]. Therefore, pulmonary cryptococcosis should be considered a possible cause of crazy-paving patterns in chest CT scans. It is necessary and crucial to carefully identify possible underlying diseases causing immunodeficiency for patients with severe cryptococcal infections.

interest to disclosure.

## Data Availability

All data and material are available for sharing if needed.

## Abbreviations

HRCT: High resolution computed tomography
RR: Respiratory rate
PAS: Periodic Acid-Schiff
CSF: Cerebrospinal fluid
MRI: Magnetic resonance imaging
HFNC: High-flow nasal cannula
ARDS: Acute respiratory distress syndrome
